# Sleep and the risk of cognitive impairment and dementia: further insights from the Caerphilly Prospective Study

**DOI:** 10.1192/bjo.2021.179

**Published:** 2021-06-18

**Authors:** James Selwood, Elizabeth Coulthard, Antony Bayer, Mark Fish, John Gallacher, Yoav Ben-Shlomo

**Affiliations:** 1 University of Bristol; 2 University of Cardiff; 3 Taunton and Somerset NHS Foundation Trust; 4 University of Oxford

## Abstract

**Aims:**

Sleep disorders are highly prevalent with growing evidence that sleep problems may predict cognitive decline and dementia. A previous analysis of the Caerphilly Prospective Study (CaPS), a cohort of middle-aged men, found that daytime sleepiness predicted vascular dementia and cognitive impairment. We have re-examined this hypothesis with additional events based on further follow-up. The study aimed to examine the role of different sleep problems in predicting cognitive impairment not dementia (CIND) and dementia. Our hypothesis was that sleep problems in mid-life would predict CIND and dementia in later life.

**Method:**

CaPS is a population cohort of men born between 1920 and 1935 and resident in Caerphilly in South Wales, first seen between 1979–1983. Cognitive tests and a sleep questionnaire were introduced at Phase III (men aged 55–69 years). The questionnaire asked about daytime dysfunction, hypnotic use, insomnia, napping, nocturnal limb movements, sleep apnoea, sleep duration, sleep latency and snoring.

At Phase V (men aged 68–82 years), poor performance on the Cambridge Cognition Exam (CAMCOG) was used to select men for detailed clinical assessment. Subjects were classified as having normal cognition, CIND or dementia. Cognitive disorders were sub-classified as vascular or non-vascular. At Phase VII (men aged 78–92 years), new cases were identified and survivors with existing diagnoses were reassessed.

We initially conducted separate logistic regressions for vascular and non-vascular cognitive outcomes with the individual sleep measures, but where there was no evidence of heterogeneity, we combined these outcomes to enhance power. We also ran ordered logistic regression models to test for association of our sleep measures with CIND and dementia from any cause, with no cognitive problems as the reference group. All models were adjusted for potential confounders such as age and lifestyle variables.

**Result:**

There were 256 cases of CIND, 155 dementia and 118 vascular cognitive disorders. 889 had normal cognition. Nocturnal limb movements strongly predicted vascular cognitive disorders (OR 2.59, 95% CI 1.34–4.98, p = 0.004). Poor sleep duration, defined as less than 6 or more than 8 hours, predicted all-cause CIND and all-cause dementia (OR 1.62, 95% CI 1.01–2.61, p = 0.045). The other sleep measures showed weak associations consistent with chance.

**Conclusion:**

We have provided further evidence that sleep problems predict cognitive decline, justifying the growing interest in sleep as a potentially modifiable risk factor for dementia. Future evidence is required from intervention studies that attempt to improve sleep parameters.

